# Actions of Brain-Derived Neurotrophic Factor and Glucocorticoid Stress in Neurogenesis

**DOI:** 10.3390/ijms18112312

**Published:** 2017-11-02

**Authors:** Tadahiro Numakawa, Haruki Odaka, Naoki Adachi

**Affiliations:** 1 Department of Cell Modulation, Institute of Molecular Embryology and Genetics, Kumamoto University, Kumamoto 860-8555, Japan; harukiodaka@ruri.waseda.jp; 2 Department of Mental Disorder Research, National Institute of Neuroscience, National Center of Neurology and Psychiatry (NCNP), Tokyo 187-8551, Japan; 3 Department of Life Science and Medical Bioscience, School of Advanced Science and Engineering, Waseda University, Tokyo 169-8050, Japan; 4 Department of Biomedical Chemistry, School of Science and Technology, Kwansei Gakuin University, Sanda City, Hyogo 662-8501, Japan; adachi@kwansei.ac.jp

**Keywords:** BDNF, neurogenesis, neural stem cells, glucocorticoids, intracellular signaling

## Abstract

Altered neurogenesis is suggested to be involved in the onset of brain diseases, including mental disorders and neurodegenerative diseases. Neurotrophic factors are well known for their positive effects on the proliferation/differentiation of both embryonic and adult neural stem/progenitor cells (NSCs/NPCs). Especially, brain-derived neurotrophic factor (BDNF) has been extensively investigated because of its roles in the differentiation/maturation of NSCs/NPCs. On the other hand, recent evidence indicates a negative impact of the stress hormone glucocorticoids (GCs) on the cell fate of NSCs/NPCs, which is also related to the pathophysiology of brain diseases, such as depression and autism spectrum disorder. Furthermore, studies including ours have demonstrated functional interactions between neurotrophic factors and GCs in neural events, including neurogenesis. In this review, we show and discuss relationships among the behaviors of NSCs/NPCs, BDNF, and GCs.

## 1. Introduction

Brain-derived neurotrophic factor (BDNF) is highly expressed in the brain, including hippocampal and cortical regions, and has pivotal roles in the maintenance of neurons in the central nervous system (CNS). Functions of BDNF through TrkB, a high affinity receptor for BDNF, and its downstream intracellular signaling pathways have been intensively studied because the BDNF/TrkB system is essential for neuronal survival and synaptic plasticity in the CNS. It is well established that activation of ERK-, Akt-, and PLCgamma-pathways are triggered by TrkB phosphorylation induced by binding of BDNF, and each pathway contributes a variety of neuronal functions, including the regulation of cell fate [1,2,3]. Recently, growing evidence has suggested that BDNF is also involved in neurogenesis, and newborn neurons contribute to the recovery process of depression-like behaviors [4,5]. Importantly, animal models of depression have demonstrated that reduced levels in both BDNF expression and hippocampal neurogenesis occurs along with depressive behaviors, suggesting that a changed status of the BDNF/TrkB system and reduced neurogenesis are associated with depressive symptoms.

Glucocorticoids (GCs) influence neuronal development and functions, as well as BDNF. Blood levels of GCs are regulated by hypothalamus-pituitary-adrenal (HPA) axis activity. It is well known that excess and chronic stress causes hyper activation of the HPA axis, which results in abnormally increased levels of GCs [6,7], and such an increased GCs has a role in the onset of mental disorders, including post-traumatic stress disorder and major depressive disorders [8,9,10,11]. Furthermore, altered neurogenesis under GC stress, especially in hippocampal region, has been an important therapeutic target to develop new drugs because suppression of hippocampal neurogenesis may contribute to the dysregulation of HPA-axis function [12,13] and is considered as one of the causes that leads to the onset of the mental disorders mentioned above.

In this review, we focus on crosstalk among BDNF, GCs, and neurogenesis. We discuss recent studies concerning functional interaction between BDNF and GCs in altered neurogenesis, and show an interesting effect of GCs: Alteration in intracellular transport of BDNF protein under GC stress.

## 2. Role of BDNF in Neurogenesis

BDNF, as one of the neurotrophins, has been intensively studied for its role in survival promotion and the regulation of synaptic plasticity in the CNS [3,14]. Importantly, positive roles of BDNF in the neurogenesis have also been demonstrated. It is considered that neurogenesis from neural stem/progenitor cells is restricted to the subventricular zone (SVZ) of the lateral ventricles and subgranular zone (SGZ) in the hippocampal dentate gyrus. In the macaque monkey brain, BDNF expression in the hippocampus and cortex (including prefrontal, temporal, and parietal association cortices) is higher than other brain regions [15]. Notably, these hippocampal and cortical BDNF expression reaches its highest level during neuronal development, especially at embryonic day 140 and the postnatal second month [15], implying the stage-specific requirement of BDNF for neuronal development and functions, including neurogenesis.

Increased neurogenesis in the granule cell layer after BDNF infusion into the hippocampus of adult rats (by implanted osmotic pumps for a month) was reported [16]. SVZ produces neuroblasts that differentiate into interneurons after migrating to the olfactory bulb (OB) in adult rodents. Interestingly, a BDNF mutant mice with a truncation of long 3’UTR of *Bdnf* mRNA, which lack dendritic expression and secretion of BDNF in hippocampal neurons [17], exhibited a decreased expression of glutamic acid decarboxylase 65, a GABA synthase, and an impaired differentiation of newborn neurons in the SGZ [18]. Moreover, the selective loss of TrkB in parvalbumin-positive GABAergic interneurons resulted in similar deficits in neurogenesis with reduced neural differentiation [18]. These results support the positive contributions of BDNF to adult neurogenesis. On the other hand, Galvao and colleagues reported that cell survival and neuronal differentiation into calbindin-positive interneurons were not influenced by loss of TrkB. They found that the grafted progenitors obtained from the SVZ of TrkB knock-out mouse could migrate to the OB in the host wild-type mice and differentiate into interneurons, suggesting that the BDNF/TrkB system is not essential for adult neurogenesis in the SVZ [19]. Because BDNF has a pivotal role in promoting survival of differentiated neurons, time- and region-specific knockdown of BDNF are necessary to reveal direct contributions of BDNF/TrkB system in neurogenesis, not in survival.

## 3. Regulation of BDNF Expression and Neurogenesis by Chemicals

Studies have shown upregulation of BDNF and increased neurogenesis occur after chemicals or neurotransmission agonist application. In animal models of depressive disorder, change in expression levels of BDNF and the rate of neurogenesis has been extensively studied. In rats that have been exposed to the chronic mild stress (CMS) to induce memory deficits and anhedonia-like behaviors, piromelatine, a melatonin and serotonin 5-HT_1A_ and 5-HT_1D_ agonist, exerted beneficial effects against memory deficits and anhedonia-like behavior [20]. Piromelatine administration reversed reduced expression of BDNF and cAMP response element-binding protein (CREB) in the hippocampus of the CMS rats, with significant recovery in the memory deficits [20]. Growing evidence indicates that selective serotonin reuptake inhibitors (SSRIs that are used in the treatment of depressive disorders) increase the expression of BDNF. Fluvoxamine, one of the SSRIs, shortened immobility time in the forced swimming test and reversed the downregulation of BDNF in the cerebral cortex and hippocampus of mice received chronic dexamethasone (an agonist for glucocorticoid receptor (GR)) infusion [21]. Chronic fluoxetine treatment also increased neurogenesis, with an upregulation of BDNF in the medial habenula and medial hypothalamus that are also considered as anxiety-associated brain regions [22]. Suppression of serotonin transporters by the RNAi technique in the dorsal raphe nucleus of mice increased the amount of extracellular serotonin, neurogenesis, and BDNF levels in the hippocampus [23]. It is very interesting whether the increased BDNF directly stimulate to produce new-born neurons or not because the activation of latent stem/progenitor cells by neuronal activity itself in the adult hippocampus has been reported [24]. Recently, an Antidepressant-Like Effect of S 47445 (8-cyclopropyl-3-[2-(3-fluorophenyl)ethyl]-7,8-dihydro-3H-[1,3]oxazino[6,5-g][1,2,3] benzotriazine-4,9-dione), a positive modulator of AMPA-type glutamate receptors, has been reported [25]. In animals received chronic corticosterone (a rodent glucocorticoid) administration and exhibited anxiety/depression-like behaviors, treatment of S 47445 reversed the depression-like behaviors and showed neurogenic effects including cell proliferation, survival promotion, and maturation of hippocampal newborn neurons. Interestingly, when neurogenesis deficient mice in which GFAP-positive progenitor cells were killed by ganciclovir treatment [26] was examined, S 47445 increased hippocampal BDNF and improved several anxiety/depression-like behaviors [25], suggesting a neurogenesis-independent process in the recovery of anxiety/depression-like behaviors. Furthermore, it has been demonstrated that Norbin, an endogenous regulator of metabotropic glutamate receptor 5 (mGluR5), promoted hippocampal neurogenesis [27]. Depressive behaviors, such as increased immobility in the forced swim test, and a significant impairment in the proliferation of NSCs/NPCs and the maturation of newborn neurons were demonstrated in Norbin-deficient mice [27]. With regard to cognitive deficits, an ameliorating effect of taurine on neurodegeneration associated with diabetes mellitus was also studied, and it could be beneficial to protect neurons and improve the learning and memory functions in a diabetic rat model [28]. Taurine is one of the abundant amino acids in the brain and contributes to the CNS function. In a depression rat model induced by chronic unpredictable mild stress, increased serum levels of corticosterone, deficits in spatial memory, and anxiety were prevented by treatment with taurine before stress exposure. Furthermore, the downregulation of basic fibroblast growth factor (bFGF), vascular endothelial growth factor (VEGF), and BDNF observed in the animal model were reversed by taurine. This report indicates regulatory functions of taurine both in the HPA-axis and in neurogenesis [29]. Indeed, it has been demonstrated that taurine improved reduced hippocampal neurogenesis during brain aging [30].

It has been reported that zinc supplementation increased the serum BDNF levels and improved depressive symptoms in obese or overweight subjects [31]. In a high-fat diet (HFD)-fed obese mouse model, a low dose of Zn^2+^ (15 ppm) reversed HFD-dependent reduction in cell proliferation, neuronal differentiation, and BDNF levels in the hippocampus, whereas a high-dose (60 ppm) enhanced the HFD-reduced hippocampal neurogenesis and BDNF levels [32]. Zinc is one of the abundant transition metals in the injured brain and was also reported to influence TrkB activation [33]. Zinc is also involved in the modulation of the spatial learning and memory function, although mice fed Zn^2+^ at high-dose (60 ppm) exhibited significant deficits in the memory and decreased levels of glutamate receptors, BDNF, and BDNF/TrkB signaling in the hippocampal region [34]. It would be interesting, therefore, to investigate whether the changed Zn^2+^ levels in injured brain affects neurogenesis via modulating the BDNF/TrkB-signaling.

## 4. Glucocorticoids and Neurogenesis

Psychosocial stress and the resultant GC response have negative impacts on the hippocampal neurogenesis and the cell fate of NSCs [35]. It is also well known that high levels of GCs caused by the activated HPA-axis in response to stressful stimuli are associated with mental disorders, including major depression [36,37,38]. Many efforts have been devoted to reveal the mechanisms by which GCs hamper neurogenesis, targeting one of the receptors of GCs, glucocorticoid receptor GR.

GR knockout mice die within a few hours after their birth because of respiratory failure due to lung immaturity. Therefore, GR heterozygous mice (GR+/−) had been use to investigate GCs and GR functions [39,40,41]. GR+/− mice (approximately 50% reduction in GR protein) develop normally without severe systemic symptoms [40]. Under basal (unstressed) condition, no difference between WT and GR+/− mice regarding depression/anxiety-like behaviors, blood levels of corticosterone, and adult hippocampal neurogenesis was observed. In contrast, under stress conditions, GR+/− mice showed dysregulation of the HPA axis activity and enhanced elevation of corticosterone levels that were induced by stress [40]. Behavioral tests revealed an increased learned helplessness in the GR+/− mice after receiving unpredictable and uncontrollable foot shocks [40]. Decreased hippocampal neurogenesis induced by stress was also exacerbated in the GR+/− mice [41]. The GR+/− mice is indeed a useful animal model of depression because their residual GR expression levels are considered to mimic the levels in severely depressed patients and their hyper-sensitivity to stressful stimuli. However, the reciprocal interaction between the expression levels of GR and the activity of the HPA axis makes it difficult to conclude whether the reduced GR expression itself and/or the elevated GCs induced by disturbance of the HPA axis alter their behaviors and neurogenesis.

To avoid the lethality at birth by systemic GR deletion, a mouse line with brain-specific deletion of GR (GRnestin-cre −/− mice) has been developed [42]. Importantly, this brain-specific GR deletion did not exhibit any obvious structural abnormality in the brain but did display hypercorticoidism- and Cushing syndrome-like symptoms, such as altered fat distribution and a reduced bone density. Interestingly, reduced anxiety under basal conditions was observed in the GRnestin-cre −/− mice. Remarkably, in spite of the elevated basal levels of plasma corticosterone, normal hippocampal neurogenesis in the GRnestin-cre −/− mice was revealed [43]. When considering the phenotypes of the GR +/− mice, targeting to reduce GR levels in the brain may not be a good therapeutic strategy because residual GR expression in GR +/− mice is sufficient to suppress neurogenesis after stress exposure, and the reduction in the GR levels can impair HPA axis functions.

We recently reported that corticosterone exposure during differentiation and maturation reduced the expression of neuron- and astrocyte-specific proteins in cultured embryonic NSCs/NPCs [44]. Corticosterone also decreased cell survival of NSCs/NPCs after differentiation, although it did not affect the proliferation of NSCs/NPCs [44]. Interestingly, knockdown of GR selectively in newborn cells in the hippocampus caused ectopic positioning of the new granule cells, and increased the synaptic contacts and basal neuronal excitability parallel with an impairment in the fear memory consolidation [45]. As expected, there is a lot of evidence concerning the negative effect of GC exposure on the neurogenesis. Anacker reported that mRNA expression of serum- and glucocorticoid-inducible kinase 1 (SGK1) was increased in the peripheral blood of patients with depression and in the hippocampus of rats exposed to unpredictable chronic mild stress [46]. They also confirmed that an inhibitor for SGK1 counteracted the reduced neurogenesis caused by GC exposure by using the hippocampal progenitor cell line. Recently, it has been reported that baicalin, a flavonoid extracted from Radix Scutellariae, improved anxiety/depression-like behavior in mouse after chronic corticosterone treatment. Oral administration of baicalin reversed the corticosterone-induced reduction in hippocampal neurogenesis and phosphorylation of SGK [47], implying a close relationship among GCs action via SGK-signaling, hippocampal neurogenesis, and depressive behaviors. Importantly, the expression of depressive behaviors and the reduction of hippocampal neurogenesis in rats appears to depend on the duration of GC treatment. Lussier compared the impacts of daily corticosterone injection for 7, 14, or 21 days, and revealed that a gradual increase in depression-like behaviors with a gradual decrease in the number of immature granule cells in the hippocampus [48]. Kott compared different methods to administer corticosterone to adult female rats, including subcutaneous injection, through the drinking water and via the implantation of pellets. The subcutaneous injection elevated serum corticosterone levels continuously while the pellet implantation induced a transient increase and no increase was observed in rats administered corticosterone via drinking water [49]. Furthermore, the only animal group injected corticosterone exhibited longer immobility time in the forced swimming test [49].

As mentioned above, GCs exert negative influences on hippocampal neurogenesis through GR activation. On the other hand, mineralocorticoid receptor (MR) to which GCs also bind functions as a positive regulator for neurogenesis. Fludrocortisone, a potent MR agonist, increased the survival and proliferation of rat hippocampal progenitor cells, and these positive effects were attenuated by high dose of dexamethasone [50], implying that MR has an effect opposite to GR in neurogenesis. Indeed, MR improves deficits in both memory formation and hippocampal neurogenesis caused by chronic early life stress (ELS) [51]. Using a transgenic mouse line with forebrain-specific MR overexpression, Kanatsou showed that limited nesting and bedding materials hampered memory formation in wildtype but not in the MR overexpression mice. They also reported an increased number of cells expressing a marker for immature neurons in the dentate gyrus of the MR overexpression mice after the ELS, as compared with wild-type mice [51]. Because corticosterone binds both GR and MR with low and high affinity, respectively, changed MR levels affects GR activation by corticosterone. It is considered that GR acts as a negative mediator but MR does as a positive one in the depression-like behaviors [52]. Therefore, to estimate the expression levels of GR and MR in normal and stressful conditions is important. The crosstalk between GR- and MR-dependent intracellular signaling would be an important issue in future studies.

## 5. Crosstalk of BDNF and Glucocorticoids in Neurogenesis

Because both BDNF/TrkB and GCs/GR systems are involved in neurogenesis, the relationship between these systems in neurogenesis is of interest. Therefore, the interaction in the neural function, including neurotransmitter release, synaptic structure has been investigated [14,53]. Many studies showed a negative influence of systemic administration of GCs on BDNF mRNA expression in hippocampal and cortical regions [54,55,56]. It has also been suggested that translation, procession, and secretion of BDNF are regulated by the GC system [57]. Hodes reported a difference in protein expression of BDNF in response to GCs between C57BL/6J and MRL/MpJ strains [58]. Chronic administration of corticosterone reduced expression of BDNF in C57BL/6J strain, but not in the MRL/MpJ strain [58], which is known to have a large regenerative capacity against the tissue injury [59]. Interestingly, the MRL/MpJ mouse strain also displayed an enhanced elevation in cell proliferation in the hippocampus and BDNF protein levels after antidepressants treatment, comparing to those of C57BL/6J mice [60]. More intimate interactions between the BDNF/TrkB and GCs/GR systems have been demonstrated. It has been demonstrated that mRNA expression of BDNF was suppressed by GC exposure via binding of GR to the regulatory sequences of the *Bdnf* gene in neuron-like cells established from mouse hippocampal cell [61]. There are several reports about the direct interaction between kinase receptors and GR to influence intracellular signaling [62,63]. We previously reported that TrkB also interacted with GR and regulated Ca^2+^ signaling mediated by PLCgamma [64]. Furthermore, phosphorylation of GR (at Ser155, Ser287, and Ser246) is induced by BDNF application for 30 min in cortical neurons [65]. Such phosphorylations of GR may be involved in neurogenesis associated with antidepressant treatment. Flavonoid baicalin administration improved the chronic corticosterone-induced depressive behaviors of mouse [47]. Baicalin treatment reversed the GR downregulation and increased phosphorylation of GR (at Ser203 and Ser211) with a significant improvement in the suppressed hippocampal neurogenesis. Interestingly, the increased expression of FK506 binding protein 51 (FKBP5) and phosphorylation of SGK1, which was caused by corticosterone were also ameliorated by Baicalin [47]. The GCs/GR system appears to affect the intracellular signaling pathways involved in the BDNF/TrkB system. Treatment with ginsenoside Rg1, one of steroidal saponins, promoted the differentiation of mouse embryonic stem (ES) cells toward neuron-like cells, which was inhibited by a GR antagonist RU486. Furthermore, Rg1 activated ERK- and Akt-signaling pathways, and the neuronal differentiation triggered by Rg1 was repressed by inhibitors for ERK- or Akt signaling [66]. Chen et al. reported that an injection of RU486 into the hippocampus disturbed the inhibitory avoidance memory formation in rats. The RU486 injection also induced significantly decreased levels of CaMKIIalpha and Akt phosphorylation in addition to a trend toward downregulation of phospho-CREB and phospho-TrkB while BDNF rescued the levels of phospho-CREB and phospho-TrkB with a rescuing trend in Phospho-Akt [67].

## 6. The Effect of Glucocorticoids on BDNF Transport

**Figure 1:**
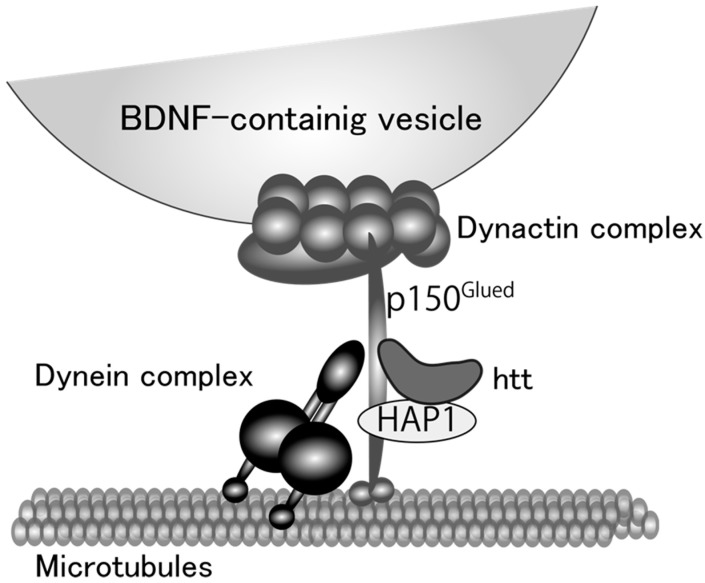
A proposed model for microtubule-dependent vesicular transport of brain-derived neurotrophic factor (BDNF) protein. Huntingtin (htt) and htt-associated protein 1 (HAP1) stabilize the linkage between dynein and dynactin complex. Dynein functions as a motor protein and dynactin complex attaches BDNF-containing vesicles to the motor protein complex with p150^Glued^ protein.

**Figure 2:**
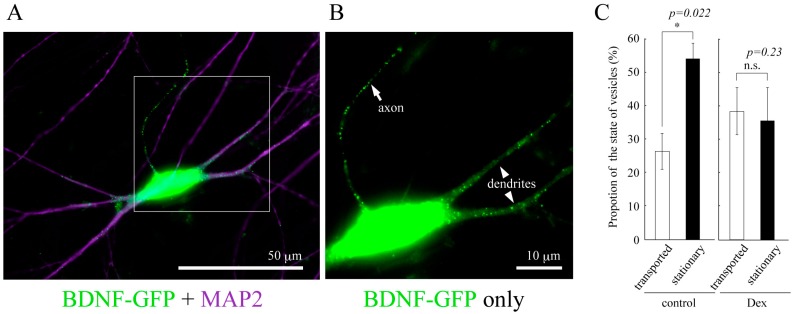
(**A**) Vesicular distribution of BDNF-GFP expressed in cultured cortical neuron obtained from rat brain (12 days in vitro). BDNF-containing vesicles are transported both in dendrites (MAP2-positive neurites) and axon (MAP2-negative one); (**B**) A magnified image of the white square region of A; (**C**) The effect of GCs on the proportion of the state of BDNF-vesicle transport. Time-laps imaging of BDNF-GFP revealed that more than 50% of BDNF-containing vesicles were not transported (stationary) in dendrites in the normal condition while DEX (an agonist for GR, 1 µM) increased and decreased the proportion of transported (>10 µm) and stationary vesicles, respectively. Data represent mean ± SE. Data was obtained from 7 (control) to 6 (DEX) neurons in four independent culture preparations including 388 and 302 vesicles, respectively. Statistical significance was evaluated by student’s *t*-test. Please see details in [78].

One of the remarkable features of neural cells is their polarized morphology. Secreted proteins in neurons, therefore, need to travel long distances in axons and/or dendrites. There are two main pathways by which neurons secrete proteins synthesized in the cell body; constitutive and regulated pathways. BDNF is preferentially secreted via the regulated pathway in which proteins are sorted into secretory vesicles, transported distally (anterograde), and proximally (retrograde) to the secretion sites, and released in response to depolarization-induced intracellular Ca^2+^ elevation, whereas NGF and NT-3 are constitutively and spontaneously secreted from neurons [68,69,70,71]. The BDNF protein is synthesized as the precursor form (pro-BDNF, 32 kDa) in the rough endoplasmic reticulum (ER) and dendrites, and many of them are secreted as the mature form (13 kDa) after receiving proteolytic cleavage in the secretory vesicles. Kinesin and dynein motor protein complexes shoulder the intracellular transport of BDNF-containing vesicles, tracking on microtubules distributed throughout polarized neurons [72,73]. Huntingtin (htt) protein also has an important role in the BDNF-containing vesicle transport. Although mutations in the htt gene results in the production of htt proteins with polyglutamine expansion (polyQ), which is causal for Huntington’s disease (HD) [74], several studies unveiled a beneficial aspect of wild-type htt protein as a positive regulator for the vesicle transport. It was showed by Saudou and colleagues that wild-type htt is involved in the motor complex, in which htt-associated protein-1 (HAP1)/kinesin and HAP1/p150^Glued^/dynactin/dynein engage in the anterograde and retrograde transport, respectively [75,76] (Figure 1). Overexpression of wild-type htt increased the velocity of vesicle transport of BDNF, and decreased the proportion of static vesicles in cultured cortical neurons, while knockdown of wild-type htt caused the opposite effects [77]. Their study clearly proved that a shortage of BDNF supply from cortical to striatal neurons caused by the diminished transport of BDNF as well as the aggregation of mutant htt is a cause of striatal neuronal loss, a common pathological feature of Huntington’s disease. These results strongly suggest an important role of normal intracellular transport of BDNF in brain function and encouraged to study a possible link between BDNF vesicle transport and GCs. We recently reported that DEX, a synthetic glucocorticoid with a high affinity for GR, promoted BDNF vesicle transport in cortical neurons through the up-regulation of htt expression [78] (Figure 2). In general, activated GR directly binds to the glucocorticoid response element (GRE) in the regulatory regions of many genes and promote or suppress their transcription [79]. Although DEX increased htt protein levels and accelerated vesicular transport through GR activation, the GRE sequence has not been identified in the regulatory region of the htt gene so far. Therefore, GR would increase intracellular htt protein levels via other signaling molecules by stabilizing htt proteins and/or mRNA or regulating its transcription and/or translation. Furthermore, Brigadski et al. revealed a change in the vesicular transport of BDNF in hippocampal neurons of an Alzheimer’s disease (AD) mouse model (5xFAD), in which amyloid β,β is abnormally increased and develops severe amyloid pathology [80]. Transport of BDNF vesicles to both directions was decreased in cultured hippocampal neurons of 5xFAD mice and in that of 5xFAD wild-type littermates treated with extracellular Aβ, suggesting a negative effect of extracellular Aβ on intracellular transport of BDNF. Impaired memory functions of the mouse model, therefore, could attribute to an early deficit in BDNF supplementation. Interestingly, Dai and colleagues also reported that cortisol (GC in human) affected the axonal transport in cortical neurons that were obtained from post-mortem human brains [81]. They found a bell-shaped dose-response for cortisol in the transport distance in both healthy humans and AD patients, although higher doses of cortisol were required for neurons of AD patients to achieve similar effects observed in that of healthy humans. When considering that synaptic plasticity and memory functions are impaired in the brains of AD patients [82], and that GCs enhance memory consolidation in which BDNF has essential roles as a synaptic modulator [83,84], these results, including ours, suggest that an “appropriate dose” of GCs may play a beneficial role for patients with HD and AD by promoting neuronal survival and memory consolidation through the enhanced neuronal transport of BDNF. Furthermore, future studies on whether the altered transport of BDNF in mature neurons under GC stress impacts on the fate of newborn neurons generated via neurogenesis are important.

## 7. Crosstalk of BDNF and Glucocorticoids in Neurodegeneration

It has been suggested that the process of neurodegeneration, as well as neurogenesis, is influenced by GCs stress [85]. Therefore, we briefly mention some of the current reports regarding an impact of BDNF and GCs on the neurodegenerative pathologies in this section. Rothman et al. reported that a triple-transgenic mouse model of AD (3xTgAD mice expressing mutant presenilin-1_M146V_, and amyloid precursor protein_Swe_, and Tau_P301L_) exhibited an elevated levels of hippocampal Aβ oligomers, increased plasma GCs, and decreased BDNF levels after chronic mild social stress, whereas such an elevated Aβ and decreased BDNF were not observed in control mice under the stress, indicating that the AD model animal is more vulnerable to the chronic stress [86]. Growing evidence suggests that a loss of neurotrophic support of BDNF may exacerbate the neuropathology in AD. It is well-known that noradrenergic neurons located in the locus coeruleus (LC) brainstem area is severely damaged in AD. Braun et al. has shown that a depletion of BDNF from a target region of the LC innervation resulted in a retrograde damage of noradrenergic neurons in an AD model mice expressing familial mutations for human presenilin-1 and amyloid precursor protein [87]. Furthermore, increased levels of mature BDNF by administrating an inhibitor for plasminogen-activator inhibitor-1 (PAI-1, an inhibitor of plasmin-dependent conversion of pro-BDNF to mature BDNF) improved cognitive function and hippocampal amyloid burden in AD model mice that were overexpressing human amyloid precursor protein [88]. In general, BDNF-dependent neurotrophic effects including survival promotion require TrkB activation via binding of mature BDNF, not pro-BDNF, the precursor form that preferentially binds to p75 receptor [89]. Interestingly, a recent study reported that exogenous application of pro-BDNF repressed the proliferation, differentiation, and migration of cultured mouse neural stem cells [90]. These studies suggest that mature BDNF has an essential role to prevent neuronal degeneration caused by Aβ toxicity. When considering that chronic GCs stress impairs the BDNF/TrkB system, excess GCs may be a risk factor for the development/progression of neurodegenerative pathologies of AD. As expected, it has also been demonstrated that depression could be a risk factor for the onset of AD [91]. Furthermore, dysregulation of the HPA axis and increased GCs in patients with Parkinson’s disease (PD) have been reported and GCs stress may contribute to the progression of the neurodegenerative process in PD [85,92]. Therefore, the decreased expression/function of BDNF in which chronic GC stress via GR activation is involved may contribute to both impaired neurogenesis and the neurodegenerative process.

Recently, natural compounds including flavonoids and small chemicals that stimulate BDNF/TrkB system have been considered as one of the candidates for therapeutic intervention [93]. Interestingly, it has been shown that increased levels of BDNF mRNA in the temporal cortex of patients with pharmacoresistant epilepsy, and the expression of GR was also influenced in the same direction [94], implying a converging behavior of BDNF and GR levels in the CNS of some neurological disorders. When considering such converging behavior, possible strategies to improve the abnormally activated GR functions should be studied carefully, although small compounds and chemicals may be beneficial tool to treat mental disorders and neurodegeneration via ameliorating the BDNF/TrkB system.

## 8. Conclusions

**Figure 3:**
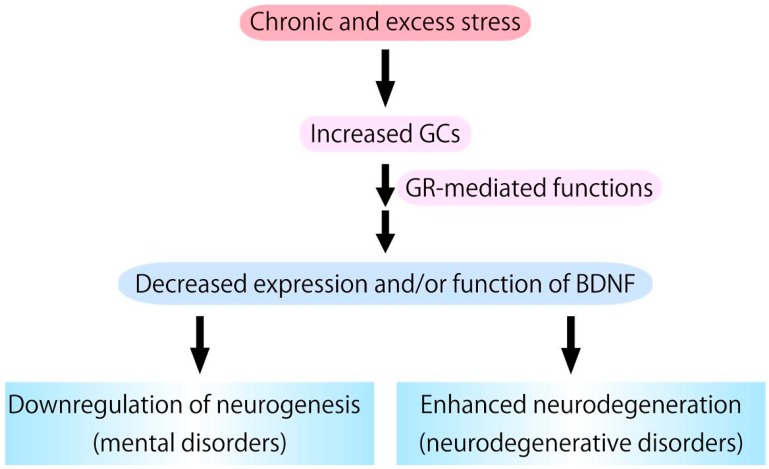
Chronic glucocorticoids (GCs) stress, which stimulates glucocorticoid receptor (GR) activation, causes downregulation of expression/function of BDNF. Because BDNF has pivotal roles in supporting neurogenesis and preventing neurodegeneration, it is possible that excess GCs functions as a negative factor in neurogenesis and a risk factor for the development/progression of neurodegenerative pathologies.

In this review, we introduced the functional interaction between the BDNF/TrkB and the GCs/GR systems in the neurogenesis. High levels of GCs seem to exert a negative influence on neurogenesis via the low affinity receptor GR-mediated function (see Table 1 and Figure 3). Recent evidence has demonstrated that activity of GR is modulated by BDNF/TrkB-related intracellular signaling, in turn, the intensive GC stress downregulates BDNF expression, although there is little information regarding whether the decreased BDNF by GC stress is indeed a direct cause for suppression of neurogenesis, or whether it is just a phenotype that is concurrently occurred. Increased GCs also exert a negative influence on neurodegenerative pathologies. Therefore, further examinations concerning the functional crosstalk between the two systems are essential to decrease the risk of neurodegenerative disease and mental disorders.

## Figures and Tables

**Table 1:** Actions of GCs on neurogenesis and BDNF expression.

In Vivo/In Vitro	Drug Application or Animals	Neurogenesis/BDNF	Reference
Adult mice hippocampus (in vivo)	GR(+/−) mice	BDNF ↓	Ridder et al. [40]
Adult mice hippocampus (in vivo)	GR nestin-cre −/− mice	proliferation -	Gass et al. [43]
Rat embryonic neural progenitor cells (in vitro)	CORT (10 µM 3–10 days)	proliferation -differentiation ↓	Odaka et al. [44]
Human hippocampal progenitor cell line (in vitro)	Cortisol (10 µM 3–10 days)	proliferation ↓ differentiation ↓	Anacker et al. [46]
Adult mice hippocampus (in vivo)	CORT (40 mg/kg daily injection for 8 weeks)	proliferation ↓ differentiation ↓	Zhang et al. [47]
Adult rat hippocampus (in vivo)	CORT (40 mg/kg weakly injection for 3 weeks)	differentiation ↓ maturation ↓	Lussier et al. [48]
Adult female rat hippocampus (in vivo)	CORT (40 mg/kg daily injection for 23 days)	differentiation ↓ *	Kott et al. [49]
Adult rat hippocampal progenitor cells (in vitro)	Fludrocortisone (1 μM 24 h)	survival ↓ proliferation ↓	Gesmundo et al. [50]
Adult C57BL/6J mice hippocampus (in vivo)	CORT (36 mg/kg/day for 7 days by pellet implantation)	proliferation ↓ BDNF ↓	Hodes et al. [58]
Adult MRL/MpJ mice hippocampus (in vivo)	CORT (27 mg/kg/day for 7 days by pellet implantation)	proliferation - BDNF -	Hodes et al. [58]
mouse embryonic stem cells (in vitro)	Ginsenoside Rg1 (10 μM, 14 days)	differentiation ↓	Wu et al. [66]

↑ increase; ↓ decrease; - no change; * Subcutaneous injection vs. pellet implantation.
